# Genomic Analysis and Antimicrobial Resistance of *Campylobacter jejuni* and *Campylobacter coli* in Peru

**DOI:** 10.3389/fmicb.2021.802404

**Published:** 2022-01-11

**Authors:** Willi Quino, Junior Caro-Castro, Verónica Hurtado, Diana Flores-León, Narjol Gonzalez-Escalona, Ronnie G. Gavilan

**Affiliations:** ^1^Laboratorio de Referencia Nacional de Enteropatógenos, Instituto Nacional de Salud, Lima, Peru; ^2^Escuela Profesional de Medicina Humana, Universidad Privada San Juan Bautista, Lima, Peru; ^3^Center for Food Safety and Applied Nutrition, Food and Drug Administration, College Park, MD, United States

**Keywords:** *Campylobacter*, whole-genome sequencing, genomic analysis, antimicrobial resistance, virulence genes

## Abstract

*Campylobacter* is the leading cause of human bacterial gastroenteritis worldwide and has a major impact on global public health. Whole Genome Sequencing (WGS) is a powerful tool applied in the study of foodborne pathogens. The objective of the present study was to apply WGS to determine the genetic diversity, virulence factors and determinants of antimicrobial resistance of the populations of *C. jejuni* and *C. coli* in Peru. A total of 129 *Campylobacter* strains (108 *C. jejuni* and 21 *C. coli*) were sequenced using Illumina Miseq platform. *In silico* MLST analysis identified a high genetic diversity among those strains with 30 sequence types (STs), several of them within 11 clonal complexes (CC) for *C. jejuni*, while the strains of *C. coli* belonged to a single CC with 8 different STs. Phylogeny analysis showed that Peruvian *C. jejuni* strains were divided into 2 clades with 5 populations, while *C. coli* formed a single clade with 4 populations. Furthermore, *in silico* analyses showed the presence of several genes associated with adherence, colonization and invasion among both species: *cadF* (83.7%), *jlpA* (81.4%), *racR* (100%), *dnaJ* (83.7%), *pebA* (83.7%), *pldA* (82.1%), *porA* (84.5%), *ceuE* (82.9%), *ciaB* (78.3%), *iamB* (86.8%), and *flaC* (100%). The majority (82.9%) of the *Campylobacter* strains carried the *cdtABC* operon which code for cytolethal distending toxin (CDT). Half of them (50.4%) carried genes associated with the presence of T6SS, while the frequency of genes associated with T4SS were relatively low (11.6%). Genetic markers associated with resistance to quinolones, tetracycline (*tetO, tetW/N/W*), beta-lactamases (*bla_oxa–61_*), macrolides (A2075G in 23S rRNA) were found in 94.5, 21.7, 66.7, 6.2, 69.8, and 18.6% of strains, respectively. The *cmeABC* multidrug efflux operon was present in 78.3% of strains. This study highlights the importance of using WGS in the surveillance of emerging pathogens associated with foodborne diseases, providing genomic information on genetic diversity, virulence mechanisms and determinants of antimicrobial resistance. The description of several *Campylobacter* genotypes having many virulence factors and resistance to quinolones and tetracyclines circulating in Peru provides important information which helps in the monitoring, control and prevention strategies of this emerging pathogen in our country.

## Introduction

*Campylobacter* is a zoonotic pathogen that causes foodborne diarrheal diseases globally (Sheppard and Maiden, 2015). *Campylobacter jejuni* and *Campylobacter coli* are the most important pathogenic species which causes approximately 90% of human infection (Newell and Fearnley, 2003). Although the most important source of infection worldwide is the consumption of undercooked poultry contaminated with *Campylobacter* or the mishandling of raw poultry products (Mullner et al., 2009), other potential sources such as wild and domestic animals have been demonstrated (Rukambile et al., 2019). The infectious dose of *Campylobacter* is 10^4^ bacterial cells (Mardones and López, 2017); however, lower infectious doses have been shown (Wieczorek and Osek, 2013), which after multiplying in the small intestine, produce inflammation with infiltration of leukocytes in the intestinal lamina, resulting in the presence of leukocytes in the stool in 25–80% of cases.

Campylobacteriosis constitutes an important public health problem due to the increase in its occurrence, new forms of transmission, increased resistance of microorganisms to antibiotics and the socioeconomic impact they cause. Furthermore, it is associated with multiple gastrointestinal conditions, including inflammatory bowel diseases (IBD), Barrett’s esophagus, and colorectal cancer (Mardones and López, 2017). In some cases, extra gastrointestinal manifestations may develop, including bacteremia, lung infections, brain abscess, meningitis, and reactive arthritis (Kaakoush et al., 2015). In some severe cases, *C. jejuni* infections contribute to post-infectious risks of acquiring neurological complications such as Guillain-Barré syndrome and Miller-Fisher syndrome (Humphrey et al., 2007), and have also been reported to lead to IBD, such as Crohn’s disease (Horrocks et al., 2009).

Few studies about the prevalence of *Campylobacter* that causing gastroenteritis are available in Peru. The most recent report indicates a 13.3% of *Campylobacter* spp. Isolated from children under 12 years (Perales et al., 2002). The frequency rate is similar in Latin American countries such as Chile, which reaches 12.7% (Valenzuela et al., 2018) or Argentina, with a value of 15.2% (Tamborini et al., 2012). On the other hand, the prevalence in animals for human consumption is much higher, reporting 16.7% of positive carcasses and 26.7% of positive cecum in Peru (Lucas et al., 2013), values even higher than them are reported in Chile and Argentina, reaching 70% of contaminated carcasses (Figueroa et al., 2009; Lopez et al., 2017).

Due to the great diversity of *Campylobacter*, the pathogenic mechanisms that cause clinical symptoms are not yet well defined (Silva et al., 2011). *cadF, racR, flaA*, and *dnaJ* genes are associated with the adherence, colonization and thermotolerance of this bacterium, and *ciaB* and *pldA* genes play a role in invasion and survival inside the host (Ramires et al., 2020; Wysok et al., 2020). Another important virulence factor is the Cytolethal distending toxin (CDT) formed by three subunits encoded by *cdtA, cdtB, and cdtC* genes, leading to apoptosis of the immune and epithelial cells of the intestine (Silva et al., 2011). Additionally, *cst-II/cst-III, cgtA*, and *cgtB* genes involved in sialic acid production are associated with the synthesis of lipo-oligosaccharides (LOS) of human ganglioside type that can induce Guillain Barré syndrome (Zhang et al., 2015). Two different type IV secretion systems (T4SS) have been described in *Campylobacter*, one codified by the plasmid *pVir*, associated with the adherence and invasion (Bravo et al., 2021), and the second present on the plasmids *pTet* and pCC31, which contribute to bacterial conjugation (Batchelor et al., 2004). Moreover, a type VI secretion system (T6SS) probably involved in virulence in *Campylobacter* is poorly understood despite its increasing prevalence in *Campylobacter jejuni* isolates (Wieczorek and Osek, 2013). T6SS plays a role in host colonization and consists of 12 central components (*TssA-TssM and tagH*) and accessory proteins codified by *hcp* genes associated with T6SS (Liaw et al., 2019).

Often the disease does not require any specific antimicrobial treatment. However, antimicrobial therapy is necessary in patients with severe and immunocompromised diseases (Wieczorek and Osek, 2013). Macrolides are the most widely used antimicrobial agents, and fluoroquinolones, tetracyclines and aminoglycosides are treatment options (Payot et al., 2006). Nevertheless, resistance to these antimicrobials in *Campylobacter* is increasing (Center for Disease Control Prevention [CDC], 2019b). The unreasonable use of antibiotics in humans and animals has contributed to increased resistance to different group of antibiotics in *Campylobacter* strains (Wieczorek and Osek, 2013). For example, a study made in a Peruvian public hospital reported 87% of strains resistant to quinolones like ciprofloxacin (Moya-Salazar et al., 2018). Also, a recent report detected 53% of animal strains resistant to macrolides (Anampa et al., 2020).

Currently, the application of WGS in the surveillance of foodborne pathogens of public health concern has become commonplace, allowing improved epidemiological surveillance and the detection of molecular markers of virulence and antimicrobial resistance in emerging genotypes (Jagadeesan et al., 2019; Su et al., 2019). This enhanced surveillance allows strengthening strategies for the prevention and control of foodborne diseases, by improving the tracking of those pathogens and targeting or eliminating the possible sources from the active commerce (Zhao et al., 2016; Painset et al., 2020). In the case of Peru, little is known of the genetic types circulating in the second decade of the twenty-first century and there is the need to increase our resolution by using a more comprehensive technique such as WGS. To test and fine tune our WGS analysis approach, and to start building our own in-country database, *Campylobacter* spp. strains from the following period (2010–2020) were selected to be subjected to WGS analysis. The aim of this study was to apply WGS analysis to determine the genetic diversity, virulence factors and antimicrobial resistance determinants of *C. jejuni* and *C. coli* populations in Peru.

## Materials and Methods

### Study Population

A total of 523 strains were obtained from 2010 to 2020 as part of the surveillance of the Laboratorio de Referencia Nacional de Enteropatogenos of Instituto Nacional de Salud, Peru, sequencing 200 strains of this group. However, only 129 good quality genomes were obtained (*C. jejuni n* = 108 and *C. coli n* = 21) and all of them were included in the analysis. Most of the strains were recovered from human clinical samples (*n* = 123) and six were recovered from poultry (*n* = 6). The number of strains from each year is indicated in Table 1.

**TABLE 1:** Number of strains of *C. jejuni* and *C. coli* used in the study by year of isolation.

Year of isolation	Strains by year	*C. jejuni* strains	*C. coli* strains
2010	9	8	1
2011	24	21	3
2012	12	10	2
2013	7	5	2
2014	8	7	1
2015	13	7	6
2016	7	7	0
2017	10	7	3
2018	7	6	1
2019	23	21	2
2020	9	9	0
Total	129	108	21

### Microbial Cultivation and Typing

The strains were cultured on blood-based agar (BD BBL™, United States) with 5% defibrinated sheep blood and incubated at 42°C for 48 h under microaerobic conditions (85% nitrogen, 10% carbon dioxide and 5% oxygen). The strains were confirmed as *C. jejuni* or *C. coli* using Lior’s biotyping scheme (Lior, 1984) and the PCR technique based on the highly conserved gene *glyA* (serine hydroxymethyltransferase) (Farace and Viñas, 2007).

### Antimicrobial Susceptibility Test

Antimicrobial susceptibility to ciprofloxacin (CIP), nalidixic acid (NA), tetracycline (TE), and erythromycin (E) was determined by agar microdilution method (Clinical Laboratory Standards Institute [CLSI], 2018). Minimum inhibitory concentrations (MIC) were interpreted according to the Clinical and Laboratory Standards Institute (Clinical Laboratory Standards Institute [CLSI], 2016), with the cutoff points being erythromycin (*R* ≥ 32μg/ml), ciprofloxacin (*R* ≥ 4 μg/ml) and tetracycline (*R* ≥ 16 μg/ml). The cut-off point for nalidixic acid (≥32 μg/ml) was established according to The National Antimicrobial Resistance Monitoring System for Enteric Bacteria (NARMS) (Center for Disease Control Prevention [CDC], 2018). *C. jejuni* strain ATCC 33560 was used as quality control of the method. The strains were cultured in Mueller Hinton Agar (BD BBL™, United States) with 5% defibrinated sheep blood and incubated at 42^°^C under micro-anaerobiosis conditions (85% Nitrogen, 10% carbon dioxide and 5% oxygen) for 24 h. Multidrug resistance was defined as the resistance of three or more classes of antimicrobials.

### Whole Genome Sequencing and Genomic Analysis

Genomic DNA extraction was performed using Pure Link Genomic DNA Mini Kit (Invitrogen, United States) and was quantified by fluorometry (Qubit 3.0 Invitrogen, Malaysia). The elaboration of the sequencing libraries was carried out using Nextera XT Library Preparation Kit (Illumina, Inc., United States), and genomic sequencing using MiSeq next generation sequencer (Illumina, Inc., United States). The quality control of each sequence was evaluated using Fastqc v0.11.5 (Andrews, 2010). Low quality adapters and nitrogenous bases were removed using Trimmomatic v0.38 (Bolger et al., 2014). The sequences were assembled *de novo* using A5-miseq v20160825 (Coil et al., 2015). The species and serotype of each strain were identified using Multilocus Sequence Typing (MLST) v2.10 (Larsen et al., 2012). To review the frequency of STs detected in this work, a minimum spanning tree (MST) was constructed using the MLST profiles obtained in this work and from other genomics studies previously carried out in Peru, using Bionumerics (AppliedMaths, bioMérieux). The reference genomes NCTC11351 (*C. jejuni*) and CFSAN054106 (*C. coli*) (Genbank ID: LN831025 and CP028187, respectively) were used to identify the chromosome contigs of each strain sequenced using Bandage v0.8.1 (Wick et al., 2015), based on BLAST algorithm with parameters of 80% identity and 20 base pairs (bp) of minimal coverage. The central genome alignment was performed using HarvestTools (Treangen et al., 2014). The maximum likelihood phylogeny was constructed using RaxML v8.0 (Stamatakis, 2014), applying the GTR + G model and 1,000 of bootstrap, defining the clades by branch length. Recombinant regions were removed by ClonalFrameML (Didelot and Wilson, 2015). The analysis of the population structure was evaluated using hierBAPS algorithm implemented in R language v3.2.3, using following parameters: (a) Include singleton SNPs, b) maximum depth = 2 and c) number of populations = 10 (Tonkin-Hill et al., 2018). The results were visualized using Grapetree (Zhou et al., 2018), associating MLST data for each evaluated species, and Microreact (Argimón et al., 2016) associating virulence and resistance genes detected.

### Identification of Virulence Factors

The detection of virulence genes was carried out using a local BLAST alignment (Altschul et al., 1990): Adherence, colonization and invasion (*cadF*, *jlpA*, *racR*, *dnaJ*, *pebA*, *pldA*, *porA*, *ceuE*, *ciaB*, *iamB*, and *flaC*), CDT (*cdtABC* operon), T6SS and T4SS genes. The prediction of coding sequences for each library was performed using Prodigal v2.6.3 (Hyatt et al., 2010). Sequence homologous genes were identified from a reference genome, with a BLAST algorithm with > 90% identity and > 60% coverage alignment with the reference. The code used for the annotation uses a Python script developed by Mestanza (2019). All the sequences obtained during this study were deposited in the GenBank database.^1^

### Identification of Antimicrobial Resistance Genes

The detection of resistance markers related to tetracycline [*tetO* and *tet(W/N/W)*], macrolides [*ermB, rplD, rplV*, and *Mef (En2)*], aminoglycosides *[APH(2″)-Iia, APH(3′)-IIIa*, and *aad(6)*], beta-lactams (*OXA-61, OXA-184*, and *cepA*), lincosamides (*LsaE*) and the multi-drug efflux pump (*cmeABC*) was performed using the comprehensive antibiotic resistance database (CARD) (Jia et al., 2017) and ResFinder 4.0 (Bortolaia et al., 2020). Point mutations such as T86I and V149I mutations in the DNA gyrase A (*gyrA*) gene associated with resistance to quinolones was checked using the output alignment of CARD and ResFinder to verify the corresponding mutation (*C. jejuni* ACA > ATA, *C. coli* ACT > ATT). Also, the A2074G/C and A2075G mutation in the 23S rRNA gene, associated with resistance to macrolides was analyzed and verified using the alignment. The results of resistance genes were associated with the previously obtained phylogeny and were also visualized using GrapeTree (Zhou et al., 2018) to evaluate the association of these genes with the genotypes found. Additionally, the correlation rate of the resistance phenotypes obtained by Antimicrobial Susceptibility Test (AST) and the genotypes by analysis was determined. Each interpretation (resistance or susceptible) for an AST result was compared to the presence or absence of a corresponding known resistance gene(s) and/or specific mutations, calculated as the sum of true positives and true negatives divided by all strains tested.

### Ethical Consideration

This study was developed within the framework of the project “Genomic Characterization of the molecular mechanisms of antimicrobial resistance and virulence factors in Clinical strains of *Campylobacter* spp. In Peru, 2010–2019,” approved by D.R. N°0154-2018-OGITT-OPE/INS. All procedures and methods were performed in accordance with ethical standards of the Declaration of Helsinki or comparable relevant guidelines and regulation. The approval of an informed consent was waived due to the retrospective nature of this study by the Institutional Committee of Research and Ethics (IRB) of the Instituto Nacional de Salud of Peru, in accordance with the national legislation and the institutional requirements for Public Health Surveillance.

### Nucleotide Sequence Accession Numbers

The draft genome sequences for all *Campylobacter* spp. Strains sequenced in this study are available in GenBank under the accession numbers listed in Supplementary Table 1.

## Results

### Determination of Susceptibility to Antimicrobials

The phenotypic antimicrobial resistance to four antimicrobial agents, determined by agar microdilution method (CLSI, VET01-S2.) is presented in Table 2. The antimicrobial resistance rate in *C. jejuni* (*n* = 108) were: ciprofloxacin (CIP) 94.4% (*n* = 102), nalidixic acid (NA) 94.4% (*n* = 102), tetracycline (TE) 71.3% (*n* = 77), and erythromycin (E) 18.5% (*n* = 20). Nineteen strains (17.59%) were resistant to all four (CIP, E, TE, and NA), while 57 (52.8%) were resistant to 3 of them (CIP, TE and NA). In the case of *C. coli* (*n* = 21), the antimicrobial resistance rate was: CIP 100% (*n* = 21), NA 100% (*n* = 21), TE 85.7% (*n* = 18), and E 52.4% (*n* = 11). Resistance to all four antimicrobials was found in 11 of the strains (52.4%), while seven strains (33.3%) were resistant to CIP, TE, and NA.

**TABLE 2:** Minimum inhibitory concentration (MIC) results observed among *C. jejuni* (*n* = 108) and *C. coli* (*n* = 21) strains.

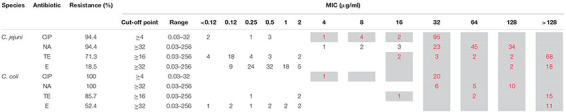

*The gray shade indicates resistance strains and red numbers indicate the number of resistance strains.*

### Genetic Diversity of Peruvian *Campylobacter* by Multilocus Sequence Typing Analysis and Minimum Spanning Tree

*Campylobacter* strains sequenced in this study (108 *C. jejuni* and 21 *C. coli*) were grouped by species. *C. jejuni* strains (*n* = 108) showed a great variety of STs. They were divided into 30 sequence types (STs) and many of them belonged to 11 clonal complexes (CC) (Supplementary Figures 1,4A). The most frequent STs were ST-2993 (*n* = 24), ST-8310 (*n* = 12), ST-6091 (*n* = 9), and ST-5789 (*n* = 7). On the other hand, *C. coli* strains were represented by 8 STs and grouped within the same CC (Supplementary Figures 2, 4B). The most frequent *C. coli* ST was ST-8317 (*n* = 4). Frequency data for each ST is detailed in Supplementary Table 2.

**FIGURE 1:**
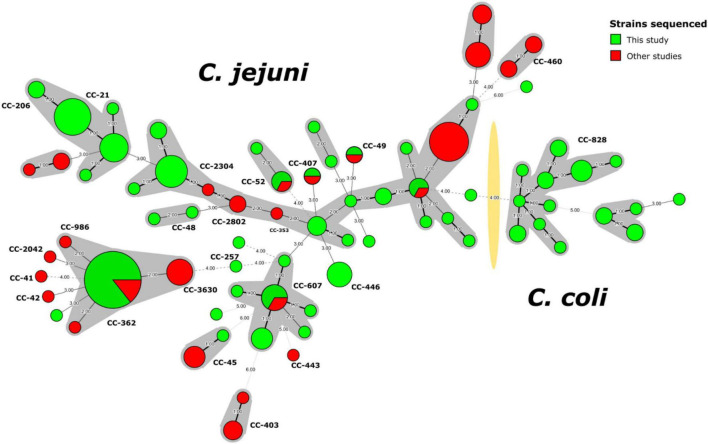
Minimum spanning tree of *C. jejuni* and *C. coli* strains sequenced in Peru. The nodes indicate the detected genotypes (STs) and are proportional to the number of strains. Clonal complexes (CC) are indicated with shadows around the corresponding nodes and with bold letters closed to the nodes. The yellow shadow divided *Campylobacter* spp. into *C. jejuni* and *C. coli*. Strains from this study (*n* = 129) are marked in green, while strains from other studies (*n* = 62) are indicated in red.

A minimum spanning tree (MST) built using the ST information for the 129 *Campylobacter* strains from this study and 62 strains from another Peruvian study (Pascoe et al., 2020; Supplementary Table 3) illustrated the high diversity of *C. jejuni* strains not previously detected in Peru (Figure 1). The presence of some new allelic profiles not previously described in the pubMLST database^2^ (Supplementary Figure 3) are highlighted, whose allelic combinations are detailed in Supplementary Table 1.

### Phylogenetic Analysis of *C. jejuni* and *C. coli*

The origin of the samples, isolation dates, WGS lineages, sequencing quality, and basic assembly metrics of all genomes are detailed in Supplementary Table 1.

**FIGURE 2:**
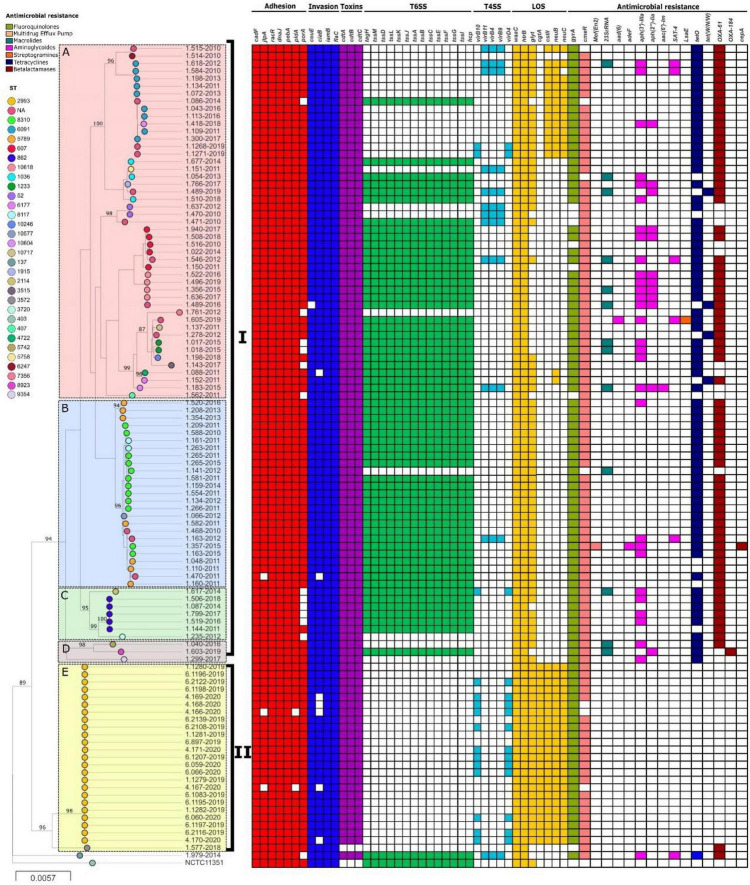
Phylogeny of *C. jejuni* (*n* = 108) constructed by maximum likelihood using strain NCTC11351 as reference. The strains are represented by circles connected by branches proportional to the phylogenetic distance. The code of each strain is indicated as a label parallel to the corresponding circles. The genotypes (ST) among the studied population are denoted by different colors. The clades are denoted by the numbers I and II. The HierBAPS populations are highlighted by colored boxes and named A–E. The presence of virulence genes is denoted by blocks in color based on six categories: Adhesion (Red), Invasion (Blue), Toxins (Purple), T6SS (Green), T4SS (Light Blue), and LOS (Yellow). The presence of resistance genes is denoted by blocks in color according to the spectrum of action: Fluoroquinolones (green), Multidrug Efflux pump (Red), Macrolides (Blue), Aminoglycosides (Pink), Streptogramins (Orange), Tetracycline (Blue) and Beta-Lactamases (Brown). The names of detected genes are indicated above the blocks. The absence of a gene is indicated by a white block.

Phylogenetic analysis using whole genomes of C. *jejuni* strains from this study showed two main clades (I and II) and 5 populations (A-E) (Figure 2), which include strains from the same CC or with little allelic variation based on the MLST. All strains from clade I were recovered in Lima. On the other hand, no geographic association was observed between strains from clade II; human strains came from seven Peruvian regions (Cajamarca, Callao, Junin, La Libertad, Lambayeque, Lima and Piura) and poultry strains came from a single region (Junin). However, most strains were isolated in 2019, belonging to an outbreak that happened that year. In fact, both human and poultry strains belong to the same genotype, ST-2993. Moreover, lineages A, B, and E appear to have a distribution according to the presence and absence of resistance and virulence determinants related to lipopolysaccharides (LOS).

**FIGURE 3:**
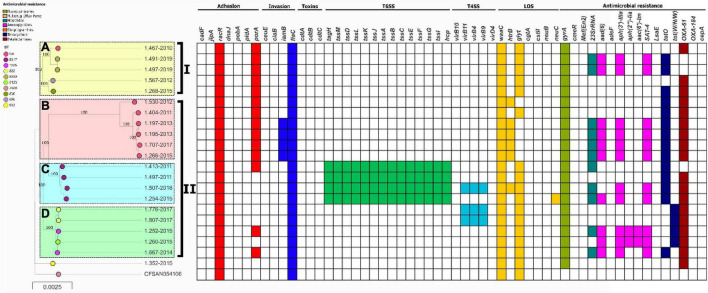
Phylogeny of *C. coli* (*n* = 21) constructed by the maximum likelihood using strain CFSAN054106 as reference. The strains are represented by circles connected by branches proportional to the phylogenetic distance. The code of each strain is indicated as a label parallel to the corresponding circles. The genotypes (ST) among the studied population are denoted by different colors. The clades are denoted by the numbers I and II. The HierBAPS populations lineages found are highlighted by colored boxes and named A–D. The presence of virulence genes is denoted by blocks in color based on six categories: Adhesion (Red), Invasion (Blue), Toxins (Purple), T6SS (Green), T4SS (Light Blue), and LOS (Yellow). The presence of resistance genes is denoted by blocks in color according to the spectrum of action: Fluoroquinolones (green), Multidrug Efflux pump (Red), Macrolides (Blue), Aminoglycosides (Pink), Streptogramins (Orange), Tetracycline (Blue), and Beta-Lactamases (Brown). The names of detected genes are indicated above the blocks. The absence of a gene is indicated by a white block.

In the case of *C. coli*, they were also grouped into two clades (I and II) which could be divided further into 4 populations with high support (Figure 3). There was no association between the lineages and the years of isolation (Supplementary Table 1). Population B is represented by strains that do not have a ST reported in the pubMLST database, and belong to an unknown not yet reported CC in *C. coli.* Strains belonging to lineage C were the only *C. coli* strains from this study carrying T6SS genes.

### Detection of Virulence Factors

*In silico* analysis for the presence of various genes associated with adherence and colonization were detected in all *Campylobacter* strains (*n* = 129): *cadF* 83.7% (*n* = 108), *jlpA* 81.4% (*n* = 105), *racR* 100% (*n* = 129), *dnaJ* 83.7% (*n* = 108), *pebA* 83.7% (*n* = 108), *pldA* 82.1% (*n* = 106), and *porA* 84.5% (*n* = 109). Presences of genes associated with cell invasion were detected: *ceuE* 82.9% (*n* = 107), *ciaB* 78.3% (*n* = 101), *iamB* 86.8% (*n* = 112) and *flaC* 100% (*n* = 129) of strains. The presence of CDT encoded by the *cdtABC* operon was detected in 82.9% (*n* = 107) of strains. T6SS encoded by *TssA-TssM, tag* and *hcp* gene were detected in 50.4% (*n* = 65) of the strains. A smaller number of *Campylobacter* strains carried T4SS genes: *virB10* and *virD4* genes in 11.6% (*n* = 15); *virB11, virB4* and *virB9* in 11.6% (*n* = 15). Additionally, genes associated with the synthesis of LOS were detected as follows: *waaC* 99.2% (*n* = 128), *htrB* 87.6% (*n* = 113), glycosyltransferase (*glyt*) 77.5% (*n* = 100), *cgtA* 18.6% (*n* = 24), *cstII* 29.5% (*n* = 38), *neuB* 31% (*n* = 40), and *neuC* 29.5% (*n* = 38). The percentage of virulence genes detected by species are detailed in Table 3 and Figures 2, 3.

**TABLE 3:** Detection of virulence and resistance genes in *Campylobacter* strains (*n* = 129) according to the studied species.

Detected genes			*C. jejuni* (*n* = 108)		*C. coli* (*n* = 21)	
			Presence	%	Presence	%
Virulence genes	Adhesion	*cadF*	108	100.00	–	–
		*jlpA*	105	97.22	–	–
		*racR*	108	100.00	21	100.00
		*dnaJ*	108	100.00	–	–
		*pebA*	108	100.00	–	–
		*pldA*	106	98.15	–	–
		*porA*	95	87.96	14	66.67
	Invasion	*ceuE*	107	99.07	–	–
		*ciaB*	101	93.52	–	–
		*iamB*	108	100.00	4	19.05
		*flaC*	108	100.00	21	100.00
	Toxins	*cdtA*	107	99.07	–	–
		*cdtB*	107	99.07	–	–
		*cdtC*	107	99.07	–	–
	T6SS	*tssA-tssM*	61	56.48	4	19.05
		*tagH*	61	56.48	4	19.05
		*hcp*	61	56.48	4	19.05
	T4SS	*virB10*	15	13.89	–	–
		*virB11*	12	11.11	3	14.29
		*virB4*	12	11.11	3	14.29
		*virB9*	12	11.11	3	14.29
		*virD4*	15	13.89	–	–
	LOS	*waaC*	107	99.07	21	100.00
		*htrB*	107	99.07	6	28.57
		*glyt*	84	77.78	16	76.19
		*cgtA*	24	22.22	–	–
		*cstII*	38	35.19	–	–
		*neuB*	40	37.04	–	–
		*neuC*	37	34.26	1	4.76
Resistance genes	Fluoroquinolones	*gyrA-T86I*	101	93.52	21	100.00
		*gyrA-V149I*	28	21.7	–	–
	Multidrug Efflux pump	*cmeABC*	101	93.52	–	–
		*Mef(En2)*	1	0.93	–	–
	Macrolides	*23S rRNA-A2075G*	13	12.04	11	52.38
	Aminoglycosides	*aad(6)*	1	0.93	10	47.62
		*adeF*	1	0.93	–	–
		*aph(3′)-IIIa*	30	27.78	11	52.38
		*aph(2″)-Iia*	12	11.11	2	9.52
		*aac(6′)-Im*	1	0.93	2	9.52
		*SAT-4*	6	5.56	11	52.38
	Streptogramins	*LsaE*	1	0.93	–	–
	Tetracyclines	*tetO*	72	66.67	14	66.67
		*tet(W/N/W)*	4	3.70	4	19.05
	Beta-Lactamases	*OXA-61*	72	66.67	18	85.71
		*OXA-184*	1	0.93	–	–
		*cepA*	1	0.93	–	–

### Detection of Antimicrobial Resistance Genes

Genes for multidrug resistance were found in *Campylobacter* strains, with a high frequency of resistance to ciprofloxacin due to a mutation in the *gyrA* gene (T86I) in 94.5% (*n* = 122), while another mutation (V149I) was detected only in 21.7% (*n* = 28). All strains with V149I mutation presented T86I mutation. In addition, the presence of the *tetO* gene, which is associated with resistance to tetracycline, was detected in 66.7% (*n* = 86). Resistance to macrolides by the A2075G mutation of 23S rRNA gene was observed in 18.6% (*n* = 24). The presence of multidrug efflux pump encoded by the *cmeABC* operon was also detected in 78.3% (*n* = 101) of the strains. Furthermore, the presence of bla_OXA–61_ gene that confers resistance to beta-lactams was observed in 69.8% (*n* = 90) of strains, as well as the presence of genes for resistance to aminoglycosides; *aph(3′)- IIIa*, *aph(2″)-Iia*, *aad(6)* and *aac(6′)-Im* in 31.8% (*n* = 41), 10.9% (*n* = 14), 8.5% (*n* = 11), and 2.3% (*n* = 3), respectively. The presence of the resistance genes described above, as well as other genes which code for beta-lactams and streptogramins, evaluated by strains and species, are detailed in Table 3 and Figures 2, 3.

### Correlation of Phenotypic and Genotypic Susceptibility

Among the strains with a quinolone-resistant phenotype (CIP, NA), 99.2% (*n* = 122) presented the mutation in the *gyrA* gene (T86I). In these strains resistant to quinolones, 77.2% (*n* = 95) presented genes of *cmeABC* multi-efflux pump operon; likewise, the presence of this operon was observed in 100% (*n* = 6) of the sensitive strains. Of the 31 strains that were erythromycin-resistant phenotypes, 77.4% (*n* = 24) had a mutation in the 23S rRNA gene (A2075G). Eighteen of these erythromycin-resistant phenotypes carried the *cmeABC* operon. The presence of this operon was also detected in 84.6% (*n* = 83) of the antimicrobial sensitive strains for erythromycin. Of the 95 strains with tetracycline resistant phenotype, 90.5% (*n* = 86) carried the *tetO* gene and 8.4% (*n* = 8) carried the *tet(W/N/W)* gene. Additionally, 77.9% of the strains resistant to tetracycline carried the *cmeABC* operon. In strains sensitive to tetracycline, the presence of this operon was detected in 79.4% (*n* = 27) of the strains (Table 4).

**TABLE 4:** Correlations of resistance phenotype and genotype in *C. jejuni* (*n* = 108) and *C. coli* (*n* = 21) strains.

Species	Antibiotic class	Antibiotic tested (AST)	No. of strains with R or S phenotype (*n* = 129)	Determinants of AMR	Presence/absence of AMR genes*	Correlation rate (%)
*Campylobacter jejuni*	Quinolones	CIP, NA	R, 102	*gyrA-T86I*	101/1	99
				*cmeABC*	95/7	93.1
			S, 6	*gyrA-T86I*	None	100
				*cmeABC*	6/6	100
	Erythromycin	E	R, 20	*A2075G*	13/7	65
				*cmeABC*	18/2	90
			S, 88	*A2075G*	None	100
				*cmeABC*	83/5	94.3
	Tetracycline	TE	R, 77	*tetO*	72/5	93.5
				*tet (W/N/W)*	4/73	5.2
				*cmeABC*	74/3	96.1
			S, 31	*tetO*	None	100
				*tet (W/N/W)*	None	100
				*cmeABC*	27/4	87.1
*Campylobacter coli*	Quinolones	CIP, NA	R, 21	*gyrA-T86I*	21/21	100
				*cmeABC*	None	100
			S, 0	*gyrA-T86I*	None	100
				*cmeABC*	None	100
	Erythromycin	E	R, 11	*A2075G*	11/11	100
				*cmeABC*	11/11	100
			S, 10	*A2075G*	None	100
				*cmeABC*	None	100
	Tetracycline	TE	R, 18	*tetO*	14/4	77.8
				*tet (W/N/W)*	4/14	22.2
				*cmeABC*	18/18	100
			S, 3	*tetO*	None	100
				*tet (W/N/W)*	None	100
				*cmeABC*	None	79.41

*Calculated as the sum of true positive and true negative divided by all tested strains.

## Discussion

In recent decades, the increase of antimicrobial resistance in *Campylobacter* has gradually become a major public health, political, social and economic problem in the world (Ramon et al., 2018). Likewise, the dramatic increase in the emergence of antimicrobial resistance observed worldwide in *C. jejuni*, specifically ciprofloxacin and tetracycline, has prompted research on the prevalence and molecular determinants of resistance. The present study is one of the largest genomic surveys of *Campylobacter* spp. Isolated in Peru. The use of whole genome sequencing (WGS) provided relevant information on the prevalence of virulence genes, antimicrobial resistance markers, as well as the phylogenetic relationships of the *Campylobacter* species and genotypes circulating in the period 2010–2020.

In this study, an important increase of antimicrobial resistance for the 129 *Campylobacter* strains was determined. During the last decade, *C. jejuni* reported 94.4% of resistance to quinolones, rates higher than reports from the National Antimicrobial Resistance Monitoring System for Enteric Bacteria (NARMS) of the United States (28%) (Center for Disease Control Prevention [CDC], 2019a). However, the results obtained here are comparable with Portugal (96.5%), Lithuania (91.5%), Spain (88.6%), Estonia (84.0%), and Cyprus (80%) (European Food Safety Authority [EFSA] and European Centre for Disease Prevention and Control [ECDC], 2019). Erythromycin resistance from *C. jejuni* also was higher (18.5%) than the United States (4.0%) (Center for Disease Control Prevention [CDC], 2019a) and European Union (2.0%) (European Food Safety Authority [EFSA] and European Centre for Disease Prevention and Control [ECDC], 2019). On the other hand, resistance level to quinolones in *C. coli* (100%) were comparable with 13 countries of the European Union (EU) for presenting extremely high levels (70.5–100%) of resistance to ciprofloxacin (European Food Safety Authority [EFSA] and European Centre for Disease Prevention and Control [ECDC], 2019). Also, resistance to macrolides obtained here for *C. coli* (52.4%) were very close to those obtained in Cyprus (12.8%). Portugal (59.5%), Malta (31.9%), and Spain (27.7%) (European Food Safety Authority [EFSA] and European Centre for Disease Prevention and Control [ECDC], 2019).

This study highlighted the wide diversity of Peruvian *Campylobacter* strains. Our findings revealed that 108 Peruvian *C. jejuni* strains were divided into 30 STs, with strains belonging to CC-362 being the most predominant (22%), followed by strains belonging to CC-21 (18%) (Supplementary Table 2 and Supplementary Figure 4). All strains from CC-362 belonged to ST-2993, which were associated with an outbreak of Guillain-Barré Syndrome in Peru (Ramos et al., 2021). Also, it is important to highlight that the frequency of this ST is very low within the PubMLST database (0.019%) (Pascoe et al., 2020). Strains belonging to CC-21 are widely distributed in the world, representing 18.9% of all strains logged to the PubMLST database. Our study reported the presence of strains in Peru belonging to CC-353, 607, and 464, which have also been found in several countries (Bravo et al., 2021; Zbrun et al., 2021), showing the transmission of these strains across very distant geographical countries, but in general, STs diversity is variable among countries, especially when comparing developed and developing countries (Noormohamed and Fakhr, 2014). Also, in this study,14 strains of *C. jejuni* that have a novel allelic profile or ST which were not previously reported in the MLST database were identified, an information corroborated by other diversity studies using MSLT, which detected usually strains that were not assigned STs (Noormohamed and Fakhr, 2014).

In the case of the *C. coli* strains, the *in silico* MLST analysis revealed that 67% (14/21) belonged to CC-828, while 33% (7/21) presented MLST profiles that were novel and not previously reported in the MLST database. The high prevalence of CC-828 strains in this study is consistent with its wide distribution throughout the world (Sheppard and Maiden, 2015; Bravo et al., 2021), being sometimes the unique CC detected by investigators (Noormohamed and Fakhr, 2014). The MST and phylogenetic analysis revealed the same composition; strains belonged to the same ST and/or CC grouped together into single clusters (Ramos et al., 2021). The reciprocity between these results is also reflected when applying tools such as Bayesian analysis (Epping et al., 2021).

Regarding the clades and populations found, previous studies have shown that it is common to find associations between clusters and the presence of resistance and virulence determinants, but not all the clades found in a phylogenetic tree are usually defined by other characteristics like geographic distribution or year of isolation, even among strains that correspond to a single country (Fiedoruk et al., 2019). In Peru, there are few previous studies of phylogenetic analysis of *Campylobacter* populations, and only one evaluated different STs Pascoe et al. (2020), which described lineages associated with the host (farm animals or humans), as well as clusters defined by their ability to cause disease (asymptomatic or symptomatic). In this study, all strains were isolated from humans and were recovered from symptomatic cases. Few strains isolated from poultry (ST-2993) grouped within the same clade (II) with other ST-2993 strains isolated from humans, complementing the study of Pascoe et al. (2020), but there is not epidemiological connection between strains of both studies. Both studies showed a wide diversity of reported genotypes in Peru, regardless of the isolation source.

The pathogenesis in *Campylobacter* is still not well defined. Several genes have been associated with the virulence of this pathogen; however, their functions are not fully understood in the development of *Campylobacter* gastroenteritis (Silva et al., 2011). In this study, a large part of the *Campylobacter* strains carried genes that have previously been associated with adherence, colonization, and invasion. The observed results for the presence of virulence genes were consistent with findings previously reported by several researchers where the *cadF, racR, flaA, dnaJ, pebA, pldA, porA, jlpA*, *ceuE, ciaB, iaB*, and *flaC* genes were also present in most of the strains that were associated with clinical cases (Ramires et al., 2020). The *cdtABC* operon, responsible for the presence of the cytolethal distending cytotoxin (CDT) in *Campylobacter*, was also present in most of the Peruvian strains from this study. Previous studies in Chile, Brazil, Korea, Poland and Ireland (Redondo et al., 2019; Ramires et al., 2020; Wysok et al., 2020; Bravo et al., 2021) have reported a high prevalence of CDT in *Campylobacter* strains isolated from patients with life-threatening diarrhea.

There are two secretion systems that have been described for *Campylobacter*: T4SS and T6SS. T4SS is encoded by genes found in the plasmid *pVir*, which are important for adhesion and invasion of intestinal epithelial cells (Wysok et al., 2020). In this study, the frequency of the genes present in *pVir* were low; these findings correlate with previous studies from Chile, Brazil, Korea, Poland and Ireland (Redondo et al., 2019; Ramires et al., 2020; Wysok et al., 2020; Bravo et al., 2021), where they also found a low prevalence of these genes, which suggests that they may not be important virulence factors in the mechanisms of pathogenesis in humans, but could facilitate the events of horizontal gene transfer that could lead to increased fitness and virulence (Redondo et al., 2019). On the other hand, the presence of genes associated with T6SS was detected in 50.4% of all *Campylobacter* strains, consistent with previously reports, which suggest that these genes might contribute to adherence, invasion and resistance to bile salts and to host cell oxidative stress (Wysok et al., 2020). Some authors connect T6SS with megaplasmids which might facilitate survival and virulence of the *C. jejuni* inside the host, enhancing hemolytic activity to lyse blood cells and then use their components as a nutrient source (Marasini et al., 2020). However, the presence of plasmids in this work was not confirmed.

The function of individual lipo-oligosaccharide (LOS) genes (*waaC, htrB, glyt, cgtA, cstII, neuB*, and *neuC*) is not very well defined; however, previous studies suggested that these genes are essential for the formation of human ganglioside-like LOS structures which can induce Guillain Barré syndrome (Zhang et al., 2015). In this study, most strains carried at least two of the genes associated with LOS biosynthesis, while all strains from ST-2993 carrying all seven genes and have been described as causing Guillain Barré in Peru in 2019 (Ramos et al., 2021).

The advantage of knowing which molecular markers for antimicrobial resistance are present in the bacterial population is crucial for the future development of prevention and control strategies to combat antimicrobial resistance in the world (Su et al., 2019; Painset et al., 2020). In our study, resistance to quinolones has generally been associated with two different markers: point mutations in the QRDR region of *gyrA* gene and the presence of *cmeABC* operon. In this study, 93.52% of *C. jejuni* and 100% of *C. coli* presented a mutation in the C257T position, which leads to the substitution of T86I in *gyrA* gene, a mutation frequently observed that confers high-level resistance to quinolones (Wieczorek and Osek, 2013; Liaw et al., 2019). On the other hand, the substitution of V149I was less frequently observed, which was according to other studies (Espinoza et al., 2020), emphasizing that only *C. jejuni* strains presented this mutation. Using the presence or absence of markers like *gyrA* could influence the clustering of strains inside a country (Leekitcharoenphon et al., 2018); however, due to the high rate of resistance strains, this observation was not possible. Besides, the *cmeABC* operon, detected only in *C. jejuni* (93.52%), encodes the most common multidrug efflux pump found in *Campylobacter* and contributes to resistance to quinolones and macrolides by decreasing the amount of the drug in cells (Bravo et al., 2021).

Regarding macrolides, the most common class of antibiotics for the treatment of *Campylobacter* infections, two markers of antimicrobial resistance used for many studies, A2075G mutation in the gene encoding *23S rRNA* and *ermB* gene (Liaw et al., 2019; Caldwell et al., 2020) were analyzed. Among the strains studied here, 12.04% of *C. jejuni* and 52.38% of *C. coli* carried the mutation of *23S rRNA* gene, which is the main marker associated with resistance to macrolides (Bravo et al., 2021). The presence of the *ermB* gene was not detected.

Currently, *Campylobacter* resistance to tetracycline is widespread in the world (Yan et al., 2006; Bravo et al., 2021). In this study, *tetO* gene, which confers resistance to tetracycline by preventing it from binding to the primary binding site on the ribosome (Wu et al., 2014), was detected in *C. jejuni* (66.67%) and *C. coli* (66.67%), data consistent with previously reported studies (Rokney et al., 2020). This represents a significant threat to public health, due to the use of antimicrobials in the poultry industry around the world, spreading drug-resistant pathogens in animals and humans.

It was also determined that *C. jejuni* (66.67%) and *C. coli* (85.71%) harbor the *bla*_*OXA*–61_ gene, which confers resistance to beta-lactams. This genetic marker has a high prevalence in *Campylobacter* worldwide (Alfredson and Korolik, 2005). The prevalence of *bla*_*OXA*–61_ has also been reported in *Campylobacter* of veterinary origin (Casagrande et al., 2020). However, beta-lactam antibiotics have limited application for the treatment of *Campylobacter* human infections (Yan et al., 2006).

Other genes detected confer resistance to aminoglycosides by enzymatic modification (Rukambile et al., 2019), which can be grouped based on their enzymatic activities: aminoglycosides phosphotransferases (*aph*), aminoglycosides nucleotidyltransferases (*ant*) also described as aminoglycosides adenylyltransferase (*aad*), and aminoglycosides acetyltransferases (*aac*) (Alfredson and Korolik, 2005). In this study, the genes *aph(3′)-IIIa*, *aph (2′)-Iia*, *aad(6)*, and *aac(6′)-Im* were detected, with the aph(3′)-IIIa gene being the most prevalent. The prevalence of these genes is variable, but *aph* and *ant* genes are the most widely described (Yao et al., 2017; Painset et al., 2020).

Additionally, resistance discrepancies between phenotypes and genotypes were observed. A strain with phenotypic resistance to quinolones did not present a mutation in the *gyrA* gene (T86I); however, the presence of *cmeABC* operon was observed, which is also associated with resistance to quinolones, macrolides and tetracyclines, so the efflux pumps would be conferring resistance to quinolones in this strain (Yan et al., 2006). Also, 7 strains with phenotypic resistance to erythromycin did not present a mutation in the gene encoding 23S rRNA (A2075G); however, the presence of *cmeABC* operon was observed in 18 strains, which would be responsible for the resistance to erythromycin in strains that did not present mutation in the 23S rRNA (Caldwell et al., 2020). Finally, 9 strains with phenotypic resistance to tetracycline did not present *tetO* gene; however, the presence of *tet(W/N/W)* gene was observed in 8 strains; likewise, the presence of *cmeABC* operon was observed in 74 strains, which would also be responsible for the resistance to tetracycline in strains that do not present *tetO* or tet*(W/N/W)* genes.

## Conclusion

In conclusion, this study highlights once more the importance of using WGS in the surveillance of emerging pathogens associated with foodborne diseases, providing genomic information on genetic diversity, possible virulence mechanisms, and determinants of antimicrobial resistance. In this study, genomic data on diversity, virulence, and resistance profiles in *C. jejuni* and *C. coli* strains circulating in Peru from 2010 to 2020 were provided. Furthermore, the reasons for a high rate of resistance to quinolones and tetracyclines observed among those *C. jejuni* and *C. coli* strains were described, as well the low rate of resistance to macrolides. The information provided in this study is of paramount importance in monitoring, control and prevention strategies to counteract the growing threat of resistance of this pathogen in our country. Finally, this study shows a possible source for the strains belonging to ST-2993 causing GBS in Peru during the past 5 years. The use of WGS for our future surveillance of *Campylobacter* circulating in the country could support the implementation of more effective One Health intervention strategies focused on the prevention and control of campylobacteriosis cases.

## Data Availability Statement

The datasets presented in this study can be found in online repositories. The names of the repository/repositories and accession number(s) can be found in the article/Supplementary Material.

## Author Contributions

WQ and RG performed the design of the work, analysis, interpretation of data, writing, and revision of the manuscript. JC-C contributed to the analysis, interpretation of data, writing, and revision of the manuscript. VH performed the experimental procedures. DF-L contributed to formal analysis and data curation. NG-E contributed to the critical revision of the article. All authors contributed to the article and approved the submitted version.

## Conflict of Interest

The authors declare that the research was conducted in the absence of any commercial or financial relationships that could be construed as a potential conflict of interest.

## Publisher’s Note

All claims expressed in this article are solely those of the authors and do not necessarily represent those of their affiliated organizations, or those of the publisher, the editors and the reviewers. Any product that may be evaluated in this article, or claim that may be made by its manufacturer, is not guaranteed or endorsed by the publisher.

## Footnotes

1
http://www.ncbi.nlm.nih.gov

2
https://pubmlst.org/
