# Supplementary material for: Genomic Analysis and Antimicrobial Resistance of Campylobacter jejuni and Campylobacter coli in Peru
Source: Front Microbiol. 2022 Jan 11;12:802404. doi: 10.3389/fmicb.2021.802404 (PMC8787162; doi:10.3389/fmicb.2021.802404)
Supplement: Supplementary file 1 [file Data_Sheet_1.PDF]

Supplementary table 2. Frequency of CC and ST of *Campylobacter* spp. (n=129)

Species	CC	ST	n
Campylobacter jejuni	CC-21	8310	12
		5789	7
	CC-45	137	1
	CC-48	6247	1
	CC-49	3720	1
	CC-52	52	2
	CC-206	8117	2
	CC-257	5742	1
	CC-353	1036	3
		1233	2
		3515	1
		4722	1
		5758	1
		6177	2
		10246	1
		10717	1
	CC-362	2993	24
	CC-446	862	5
	CC-607	607	5
		1915	1
		10618	4
	No CC assigned	407	1
		2114	1
		3572	1
		6091	9
		7356	1
		8923	1
		9354	1
		10577	1
		10604	1
		NA	13
	SUBTOTAL		108
Campylobacter coli	CC-828	825	2
		830	1
		860	1
		902	1
		1055	2
		5123	1
		8317	4
		8939	2
	No CC assigned	NA	7
	SUBTOTAL		21
TOTAL			129
