# Supplementary material for: Genomic Analysis and Antimicrobial Resistance of Campylobacter jejuni and Campylobacter coli in Peru
Source: Front Microbiol. 2022 Jan 11;12:802404. doi: 10.3389/fmicb.2021.802404 (PMC8787162; doi:10.3389/fmicb.2021.802404)
Supplement: Supplementary file 2 [file Data_Sheet_2.PDF]

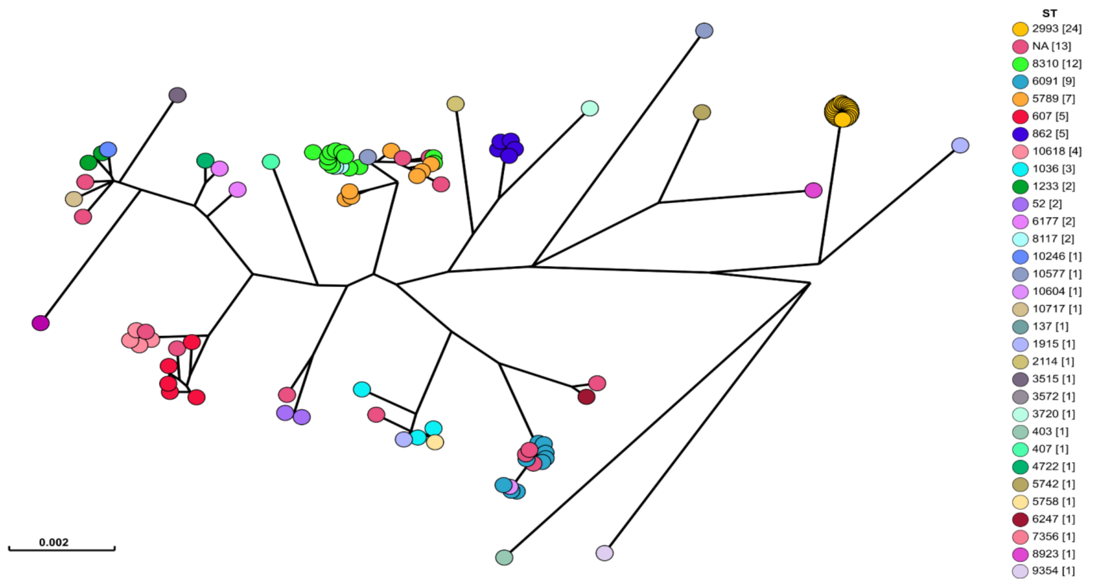

Supplementary Figure 1. Central genome-based phylogeny of *C. jejuni* (n=108) using NCTC11351 strain as reference. The minimum spanning tree for *C. jejuni* is based on analysis of 102 clinical strains and 06 from poultry. The strains are represented by circles connected by branches proportional to the allelic distance. The genotype distribution among the studied population is denoted by color.

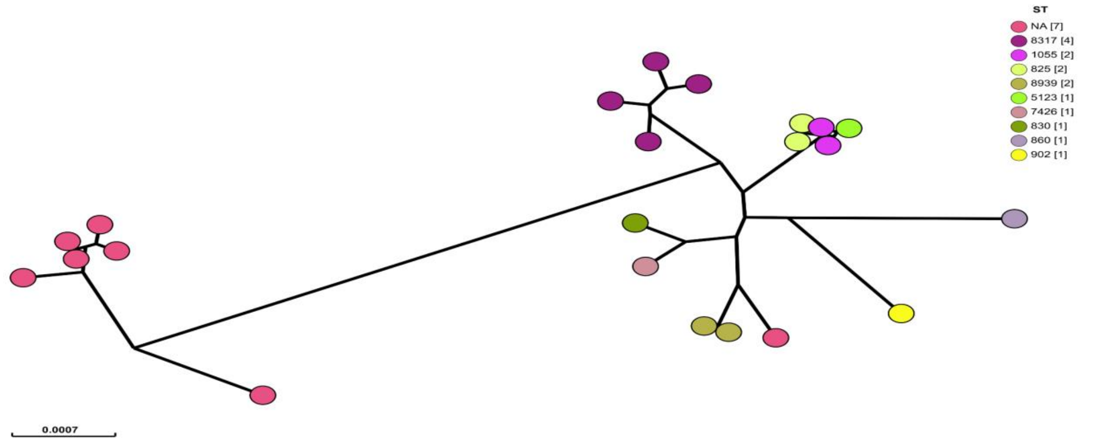

Supplementary Figure 2. Central genome-based phylogeny of *C. coli* (n=21) using CFSAN054106 strain as reference. The minimum spanning tree for *C. coli* is based on analysis of 21 clinical strains. The strains are represented by circles connected by branches proportional to the allelic distance. The genotype distribution among the studied population is denoted by color.

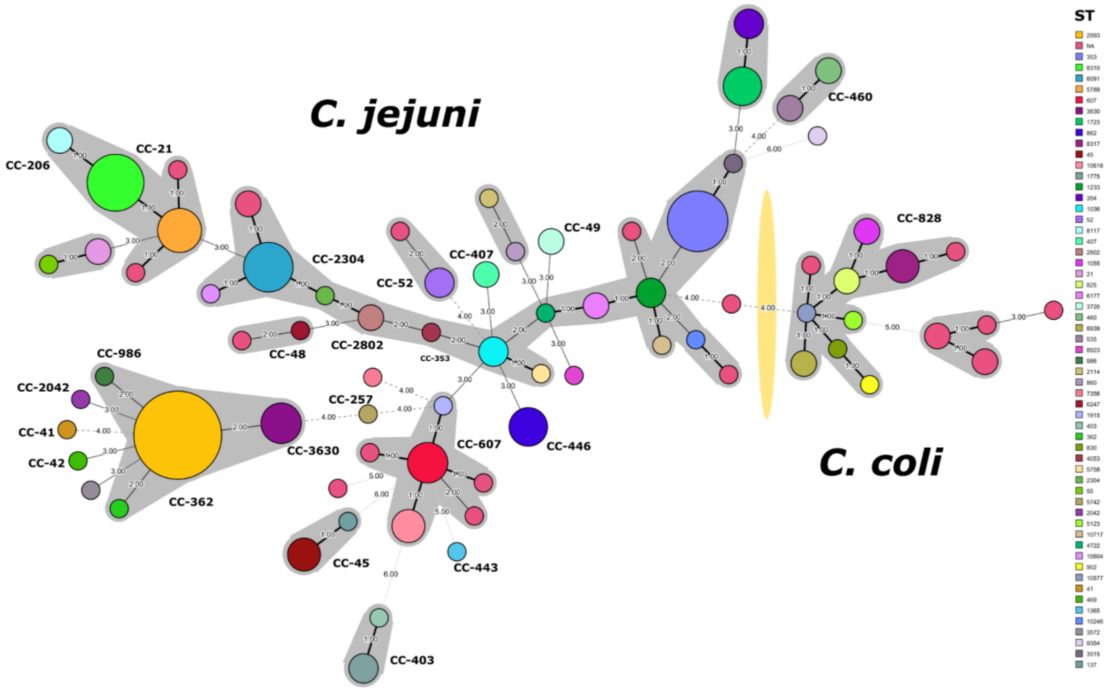

Supplementary Figure 3. Minimum spanning tree of *C. jejuni* and *C. coli* strains sequenced in Peru. The genotype distribution (ST) among the studied population is denoted by color. Clonal complexes (CC) are indicated with a gray background. The nodes are proportional to the number of strains they include. Allelic differences are indicated by numbers on the branches.
