# Supplementary material for: Genomic Analysis and Antimicrobial Resistance of Campylobacter jejuni and Campylobacter coli in Peru
Source: Front Microbiol. 2022 Jan 11;12:802404. doi: 10.3389/fmicb.2021.802404 (PMC8787162; doi:10.3389/fmicb.2021.802404)
Supplement: Supplementary file 3 [file Data_Sheet_3.PDF]

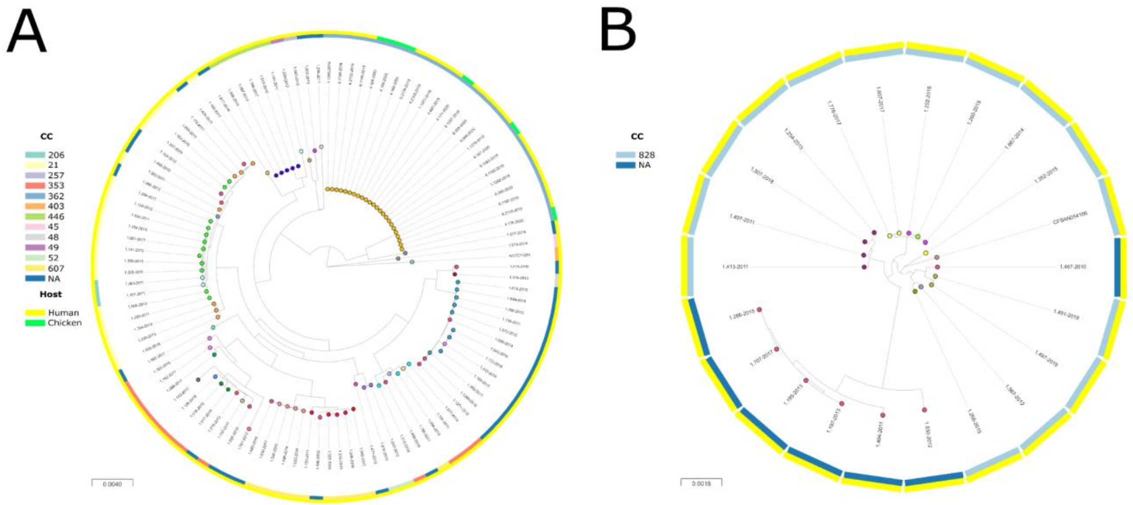

Supplementary Figure 4. Genetic diversity of *Campylobacter* spp. A) Circular phylogeny of *C. jejuni* constructed by maximum likelihood including 108 Peruvian strains (102 clinical and 6 from poultry) using the strain NCTC11351 as reference. **B)** Circular phylogeny of *C. coli* constructed by maximum likelihood including 21 clinical strains using the strain CFSAN054106 as a reference. The code of each strain is indicated as a label parallel to the corresponding circles. The genotypes (ST) among the studied population are denoted by different colors. The clonal complexes (CC) are highlighted on the inner ring, while the host from which each strain was recovered is indicated on the outer ring.
